# Sheep enteric cestodes and their influence on clinical indicators used in targeted selective treatments against gastrointestinal nematodes

**DOI:** 10.4102/ojvr.v86i1.1648

**Published:** 2019-02-28

**Authors:** Salah Meradi, Jacques Cabaret, Bourhane Bentounsi

**Affiliations:** 1 Institute of Veterinary Sciences and Agronomic Sciences, University of Batna 1, Algeria; 2 National Institute for Agricultural Research (INRA), François-Rabelais University, France; 3 Institute of Veterinary Sciences, University of Constantine 1, Algeria

## Abstract

Clinical indicators such as diarrhoea (DISCO) or anaemia (FAMACHA^©^) are used as a measure for targeted selective treatments against gastrointestinal nematodes (GIN). Enteric cestodes such as *Moniezia* may interfere directly with DISCO or indirectly with the FAMACHA^©^ score. We investigated 821 Ouled Djellal rams naturally infected in a steppe environment (GIN alone, cestodes alone, GIN and cestodes) or not. The rams were treated with ivermectin 2 months before being slaughtered to reduce the impact of nematodes on the clinical scores; however, persistent or newly acquired GINs were not related to both scores. Of the non-infected rams (*n* = 296), 26% identified as needing treatment against GIN using the FAMACHA score, and 34.5% using DISCO would have been thus selected. This implies that the clinical indicators used for the targeted selective treatment of gastrointestinal nematodes are not fully reliable when a low infection is recorded and may well be influenced by confounding factors. As expected, only DISCO was affected by cestode infection, and we suggest that the presence of *Moniezia* should also be taken into consideration.

## Introduction

A wide diversity of opinions exists regarding pathogenicity of cestodes in sheep. Cestodes from the genus *Moniezia* are regarded as more pathogenic in young lambs, whereas other cestode genera also recorded in adult sheep are either considered less pathogenic (*Avitellina*) or virtually non-pathogenic (*Stilesia*) (Soulsby 1982). Studies by Elliott (1984) did not consider *Moniezia* as a cause of diarrhoea in sheep. Conversely, Cabaret et al. (2006) found a relationship between diarrhoea in lambs and the presence of *Moniezia*. There is thus a real need to evaluate the influence of cestodes on sheep health and production, especially as they have marked their concomitant presence with gastrointestinal nematodes (GIN) in steppe regions in Eastern Algeria (according to unpublished data from our laboratory). Owing to the spread of anthelmintic resistance in GIN, targeted selective treatments are being undertaken only for animals identified as sick to reduce the use of anthelmintics. This is based on the fact that in a flock, the populations of gastrointestinal parasites in small ruminants are highly aggregated and dispersed (Gaba, Ginot & Cabaret 2005). Performance indicators such as body weight (Cottle 1991; Stafford, Morgan & Coles 2009), milk production (Hoste et al. 2002) or the use of clinical indicators, such as anaemia score using the FAMACHA^©^ system (Van Wyk & Bath 2002), dag score (Larsen et al. 1994) and the diarrhoea score DISCO (Cabaret et al. 2006) were utilised for GIN. DISCO was found to be the most relevant indicator in the steppe regions of Eastern Algeria and Morocco (Bentounsi, Meradi & Cabaret 2012; Ouzir et al. 2011), where *Teladorsagia circumcincta, Nematodirus* and *Marshallagia marshalli* were the most prevalent species, and *Haemonchus contortus* was occasionally reported (Bentounsi, Meradi & Cabaret 2012; Meradi et al. 2011; Ouzir et al. 2011). FAMACHA^©^ scores and body weight have not been reliable indicators for detecting lambs in need of treatment in our conditions (Bentounsi, Meradi & Cabaret 2012). The clinical impact of enteric cestodes is poorly known and could bias the selection of sheep to anthelmintic treatment against GIN. It was evaluated in this paper using clinical indicators of diarrhoea (DISCO) and anaemia (FAMACHA^©^) obtained before slaughter of selected rams previously treated with ivermectin to also eliminate, in part, the bias owing to GIN. It was expected that the diarrhoea score would be influenced and that the FAMACHA^©^ score would remain unchanged. The evaluation of these hypotheses was undertaken in rams in Eastern Algeria.

## Materials and methods

### Study site and experimental animals

This study was carried out between November 2015 and February 2016 in sheep slaughtered at Batna abattoir in Eastern Algeria. Experimental animals were rams aged between 12 and 14 months, the accepted age for slaughter. The rams investigated in the present paper are of Ouled Djellal breed, grazed under an extensive system in the region of Batna, which is characterised by a steppe climate. It is local practice to treat animals with subcutaneous injections of ivermectin 2 months before slaughter although they remain on their usual pastures. In our study, only treated rams were selected. A total of 821 rams were examined.

### FAMACHA© and DISCO investigations

The FAMACHA^©^ system is based on a semi-quantitative evaluation of the eye mucosal colour, which is classified into one of five categories on a colour chart, indicating a level of anaemia; 1 (red, non-anaemic) to 5 (white, severely anaemic) (Van Wyk & Bath 2002). The DISCO indicator categorises a faecal sample according to the following consistency index; 1 (corresponds to normal sheep faeces in pellets), 2 (corresponds to ‘soft’ faeces [similar to cow pat]) and 3 (corresponds to diarrhoea [semi-liquid faeces]). These scores correlate to 40%, 25% and 15% dry matter in faeces, respectively (Cabaret et al. 2006). These indicators (FAMACHA^©^ and DISCO) and faecal samples were taken from each animal in lairage at the slaughterhouse. Animals under transport stress, with discomfort or that had conjunctivitis were excluded from scoring and faecal sampling.

### Parasitological investigations

Faecal samples were collected per rectum to estimate GIN. The gastrointestinal infection intensity was estimated by the number of nematode eggs per gram of faeces (EPG) using a modified McMaster method with a saline solution (1.18 specific gravity) sensitive to 15 EPG (Raynaud 1970). In addition, a flotation method was performed at a sensitivity of 7.5 EPG (Raynaud 1970) when the McMaster result detected no eggs. The eggs were identified to genus level only, as *Marshallagia* and *Nematodirus*, and other GIN. After slaughter, the small intestine was washed and the content was filtered through a sieve (250 *µ*m mesh). Cestodes were collected and transported to the Parasitology Laboratory, where the scoleces were counted and proglottids examined. The number of scoleces was used as an estimation of the number of parasites. Samples from strobila of each cestode were taken and preserved in 10% formalin. Cestodes were examined on a slide with cover glasses under a light microscope. The proglottids, after clearing and staining with acetic carmine, were identified on morphology (Soulsby 1982). Prevalence (% of ram infected) and abundance (average number of worms among rams) were calculated (Bush et al. 1997).

### Statistical analysis

The data were analysed using the statistical package SPSS version 20. The univariate analyses of variance were performed to assess the significant differences in the FAMACHA^©^ score and DISCO in relation to infection groups. Non-parametric Spearman coefficient of correlation (r_s_) between clinical indicators and infection were also calculated.

### Ethical considerations

The experiment was approved by the Local Ethical Committee for Experimental Animals in Algeria and was conducted in compliance with the universal ethical standards.

## Results

### The helminth fauna

The overall prevalence of cestodes in rams studied in the region was 9.9%. The cestodes in decreasing order of prevalence were *Moniezia expansa* (5.18%), *Avitellina centripunctata* (3.35%), *Stilesia globipunctata* (0.97%) and *Moniezia benedeni* (0.36%). The prevalence of GIN was 57.1%. The EPG was low (see Table 1). Four parasitological groups could be established: no infection (296 rams), cestode infection only (32 rams), nematode infection only (444 rams) and cestode and nematode infection (49 rams) (see Table 2).

**TABLE 1:** Distribution of gastrointestinal nematode faecal egg counts in an abattoir (821 rams) in Eastern Algeria showing faecal egg count, prevalence (%P) and nematode categories.

Faecal egg count	Nematode categories*		
	*Marshallagia*	*Nematodirus*	Other gastrointestinal nematodes
7	9.5	10.1	15.8
15	1.8	2.7	6.0
30	1.8	1.5	2.2
45 and over	1.0	1.1	0.9

* , Prevalence (%P).

**TABLE 2:** Clinical indicators in relation to parasitological groups of rams indicating mean abundance ± standard deviation (MA ± SD).

Parasitological groups (no. of rams)	Clinical indicators	
	FAMACHA^©^ (MA ± SD)	DISCO (MA ± SD)
No infection (296)	1.90 ± 0.80	1.40 ± 0.59
Cestode infection only (32)	1.84 ± 0.13	1.91 ± 0.89
Nematode infection only (444)	1.68 ± 0.75	1.42 ± 0.62
Cestode and nematode infection (49)	1.82 ± 0.81	1.88 ± 0.83

MA, mean abundance; SD, standard deviation.

### Clinical indicators (FAMACHA^©^ and DISCO)

The FAMACHA^©^ scores extended from 1 to 4 and were found in 42.9%, 37.8%, 18.4% and 0.9% of the rams, respectively. The DISCO values (from 1 to 3) were 62.7%, 28.5% and 8.8% of the rams, respectively.

### Relationship between type of infection and the two clinical indicators

DISCO was significantly (*p* = 0.00l) lower in the non-infected group and in the group with nematode infection only, and significantly (*p* = 0.0001) higher in the group with cestode infection only. In the group harbouring cestodes and nematodes, DISCO correlated with the number of cestodes (*rs* = 0.67; *p* = 0.0001) but not with nematode EPG (*rs* = 0.25; *p* = 0.09). The group harbouring nematodes only had a significantly lower FAMACHA^©^ score (*p* = 0.003) (see Table 2).

### Clinical indicators could select the false-positive rams for treatment

Seventy-seven rams in the non-infected group (296 rams) had a FAMACHA^©^ score ≥ 3, which is often taken as an indication for treatment against GIN; however, no infection could be detected. DISCO 2 or DISCO 3 values indicate necessity for treatment. DISCO 2 or DISCO 3 were found in 86 and 16 rams, respectively, from a total of 296 rams. By using clinical indicators as the only indication of a nematode infection, 77 and 102 rams would have been incorrectly treated as false positives.

## Discussion and conclusion

This low prevalence of enteric cestodes seems to be related to the steppe climate (cold semi-arid) of the region, which is unfavourable for the development of oribatid mites, intermediate hosts of cestodes (Denegri 2001). It is also probably because of the age of the sheep slaughtered (Soulsby 1982), as they were slightly over 1 year of age. Similar prevalences within sheep of similar age groups have also been reported in Turkey (4.43%), according to Aydenizoz and Yildiz (2003), and in several regions in Eastern Algeria such as El Oued (13.2%), Constantine (13.3%) and Mila (12.1%) (Bentounsi & Meradi 2016). The very low infection rate of GIN is related to the treatment given earlier: the rams are treated mainly with ivermectin 2 months before slaughter. Nevertheless, they remained infected as some GIN are resistant (Bentounsi et al. 2007) or they become re-infected. Use of these clinical indicators, designed to detect high infection levels, could leave low infections undetected.

In the present study, DISCO was associated with cestode infection as previously shown for *Moniezia* by Cabaret et al. (2006). As expected, the clinical indicator, FAMACHA^©^ score, was not influenced by cestode infection and thus remains a good indicator for some GIN (mainly *Haemonchus* sp.) whatever the infection with cestodes. DISCO is possibly a more general clinical indicator, being susceptible to vary to different pathogens (coccidia, GIN or *Moniezia* among parasites), and thus requires parasitological diagnostics to be more effective in managing gastrointestinal tract infections.
